# P312: Blood safety: how to prevent contamination by HIV, Hepatitis B and C and Syphilis to blood donor in Lubumbashi

**DOI:** 10.1186/2047-2994-2-S1-P312

**Published:** 2013-06-20

**Authors:** O Wembonyama, ML Tshilolo, C Tshintshiompo, Mpoy WC, Watu MC

**Affiliations:** 1 University of Lubumbashi, Lubumbashi, Congo, The Democratic Republic of the; 2 CEFA, Munkole Hospital, Kinshasa, University Mbujimayi, Mbujimayi, Kinshasa, Congo, The Democratic Republic of the; 3 University Institute of Congo, Lubumbashi, Congo, The Democratic Republic of the

## Introduction

Blood transfusion is an essential part of healthcare requiring equitable access to safe blood. In DR Congo, millions of patients do not have timely access to safe blood for lack of blood donors with reliable risk products contaminated with HIV, hepatitis and other chronic infections.

## Objectives

To study the prevalence of HIV, HBV, HCV, RPR among blood donors and answers to reduce the risk of viral transmission by blood.

## Methods

A descriptive study of serum markers cross of HIV / AIDS, Hepatitis B and C and syphilis among the blood donors (volunteer and paid family) admitted to provincial blood center in the province of Katanga in 2007.

## Results

It was observed in blood donors. A seroprevalence:

- 6.5% for HIV among volunteers against 4.8% for family donors and 2.2% for paid donors.

- 3.9% for HBV among volunteers against 5.5% for the family, and 2.7% for wages.

- 0.45% for HCV among volunteers against 0.94% to 0.67% and the family paid for.

- 0.00% for the RPR among volunteers against 0.11% to 0.07% and the family paid for.

Resistance to the implementation of standards related to the transfusion'' insufficient information caregivers, blood banks and transfusion safety and quality management, as well as the disruption of health care system and the multiplicity of indications and emergencies.

## Conclusion

Need for screening of antibodies on blood donation and transfusion safety strengthen these four infectious diseases.

## Disclosure of interest

None declared

